# Prevention, precision prevention and precision medicine

**DOI:** 10.1093/exposome/osaf011

**Published:** 2025-10-25

**Authors:** Paolo Vineis

**Affiliations:** 1 School of Public Health, Imperial College, London, United Kingdom

**Keywords:** precision prevention, NNT, healthcare costs, individual responsibility, expotype, nanny-State

## Abstract

It has been proposed that the measurement of the sequence of exposures an individual is exposed to over time (or “expotype,” complementary to the genotype) would be a way to promote precision medicine at the patient’s bed, and also primary prevention. The incorporation of new technologies, like omics, genotypes, Electronic Health Records, georeferencing and AI, into public health is attractive; however, the thesis of this Commentary is that the use of the exposome approach for precision prevention needs to be examined critically. The use of the expotype for practical purposes requires proof of causality, and the added value of the expotype may be limited, for example if measured through the NNT (Number Needed to Treat). In addition, the medical system may not afford the extra budgets to measure the expotype at the patient’s bed (particularly if it goes beyond the anamnesis and georeferencing, and includes omic measurements). I also argue that public health is largely a matter of structural interventions at the societal level, like taxation, and not only of individual responsibility. The main successes in tackling diseases have been tobacco taxation, sugar taxes and of course vaccination, rather than individualized health promotion. The proposal of extending precision therapy to precision prevention should not divert our attention from the great opportunities for prevention at the population level. Population prevention is cheaper, it usually addresses several diseases with a single intervention (think of smoking or air pollution) and does not need to be replicated at each generation like cure.

In the context of exposome initiatives and papers, it has been proposed that one of the goals of the exposome is to transfer research into the practice of precision prevention or precision public health.^1^ The term is borroughed from precision medicine. According to one definition, “Precision medicine is a revolutionary approach for disease prevention and treatment that takes into account individual differences in lifestyle, environment, and biology,”^2^ or also “precision medicine aims to deliver the right intervention to the right patient at the right time.” Precision prevention or precision public health consist in profiling individual exposures in their totality, possibly according to a life-course approach, to allow more individualized assessment of risks and therefore individualized prevention. The concept of “expotype”—defined as the vector of exposures an individual is exposed to over time—has been proposed in addition to the genotype and the phenotype.^3^ More specifically, the genotype and the expotype combine together to lead to the phenotype, ie, genetic susceptibility and the multiplicity of exposures in the life-course combine to lead to health and disease. The incorporation of new tehcnologies, like omics, genotypes, Electronic Health Records, georeferencing and AI, into public health is attractive; however, a number of questions arise in relation to transfer into practice. Though the exposome approach is clear as a research program, its applications in public health are still blurred. A clear distinction between research and practice is needed.

## The value of the exposome in the clinic

Clinical medicine refers to the treatment of a diseased person or a person with signs or symptoms. The exposome can contribute to this in several ways, for example by identifying chemical exposures that interfere with the metabolism and effectiveness of pharmaceutical drugs; or by improving diagnosis through the identification of an environmental exposure involved in the etiology of a certain disease. For example, some phthalate plasticizers are responsible of receptor-based induction of the drug-metabolizing enzyme CYP3A4, thus interfering with the action of drugs that undergo the same metabolic pathway. An example of the contribution to improving diagnosis is discovering that a patient with a liver mass has been exposed to vinyl chloride, which can orientate toward the diagnosis of hemangiosarcoma of the liver. By providing a profile of exposures and perhaps blood measurements of chemicals and their impacts on biomarkers, the expotype can help refine diagnosis and monitor the clinical course. After all, biomarkers such as triglycerides, glucose, cholesterol, etc, are not only meant to predict and monitor diseases, but also to infer the impact of exposures like dietary behaviors. In the case of cardiovascular diseases, risk charts have been developed that allow the clinician to predict risk. They correspond broadly to an “expotype,” since they include eg, physical activity, BMI and indirect measures of dietary habits like triglycerides and glucose. These charts are used by clinicians to support health promotion by suggesting preventive practices (or administering drugs like statins and GLP-1 receptor agonists) to high risk patients. The question is, how can—along the same lines—the measurement of the chemical expotype (with or without omic measurements eg, via mass spectrometry) help the clinician?

## The value of causality assessment

We can imagine different scenarios here:

1. In the context of the expotype characterization, a chemical is found in the blood of a patient with a disease, let’s suppose an autoimmune disease. Is it a known immunotoxicant, for example a PFAS? And if so, what is the probability that it is causally involved in the disease? PFAS are in the blood of almost the totality of the population, though one can imagine that particularly high levels can ring a bell. Would the identification of high levels of PFAS change the therapy? Would it be useful to initiate a primary prevention pathway (which, most likely, would consist in societal and collective action)?
2. If the chemical is not known to be causally relevant, what is the utility for prevention? The expotype is not supposed to help if a causal association with disease, or at least its predictive ability, has not been demonstrated yet. The expotype is likely to express multiple exposures, most of which have not been causally associated with disease onset yet, ie we are still in the domain of discovery, not of prevention.

Apart from the context of discovery (research), a possible interpretation is that the expotype is a combination of known causal factors that interact with each other within causal constellations (“Rothman’s pies”), something that is not new. The model can be the risk chart for cardiovascular diseases, that can be mimicked by the expotype. Several CVD risk charts are available.^4^ They assess usually the likelihood of experiencing a major cardiovascular event (myocardial infarction or stroke) in a given time period, based on the values of multiple risk factors like gender, diabetes, smoking, age, systolic blood pressure and total serum cholesterol. Beyond a certain level of risk, preventive advice is given (eg, smoking, diet, physical activity), and if this fails a treatment is started, for example with statins. It is a fact that physicians often start treatment with statins even in the absence of behavioral modifications, ie, pharmaceutical treatment is preferred to primary prevention.

## The value of the number needed to treat

The benefit of the treatment, either preventive or pharmaceutical, is estimated with the Number Needed to Treat, ie, how many people need to undergo a specific intervention to prevent one cardiovascular event. For example, if the NNT for statins is 217, it means 217 people need to be treated with statins to prevent one non-fatal heart attack.^5^

This is not the context to judge whether 217 is a large or a convenient number; it needs to be complemented also by the number needed to harm (NNH). For most interventions based on screening of the population with a test (and the expotype would correspond to a form of screening, like measuring cholesterol in an apparently healthy individual), NNSs (Numbers Needed to Screen) tend to be large. In one study, in the first 8 years from the start of each screening program, the NNS to prevent one death from breast cancer was 781 women and the NNS to prevent one death from colorectal cancer was 1250 people.^6^ For a long time, attempts have been made to increase the accuracy of screening tests with the addition of biomarkers, but success has been limited.

At least two questions arise:

a) Does the measurement of the expotype (that we suppose to be predictive of the disease onset) contribute to decrease the NNT, NNS or increase NNH?
b) Does such measurement influence therapeutic or preventive choices?

Answers need to be found for such questions before any proposal of an expotype is made.

These questions were already raised for genetic screening many years ago.^7^

In practice, it seems that with precision prevention we easily fall into one of the main problems of medicine nowadays, that is the disproportion between measurement/diagnostic ability and the solutions—including therapies—that are available.

## The value of exposome in primary prevention

Primary prevention is avoidance or reduction of exposure. As Geoffrey Rose pointed out a long time ago,^8^ a large number of people at a small risk may give rise to more cases of disease than the small number who are at a high risk. In other worlds, there is a gap between individual and population benefits. Rose called it the prevention paradox: “A preventive measure which brings much benefit to the population offers little to each participating individual.” This idea is captured well by the concept of NNT, since this indicator depends on the intrinsic efficacy of the intervention and on the frequency of the outcome. For a frequent outcome, the NNT will be lower, ie, fewer individuals will need to be treated to obtain a success. This explains the advantage of limiting the intervention to high-risk individuals, because the frequency of the outcome is higher among them.

The use of biomarkers (including genetic tests and in principle the expotype) for the selection of high-risk individuals may thus make sense. A focus on high-risk individuals because of their genetic background has been used successfully: an example is screening for phenylketonuria in newborns, which permits simple dietary preventive actions. However, other examples do not seem to be persuasive. One case is the proposal to select smokers who have the greatest difficulty in stopping, to treat them in a more pharmacologically aggressive way. The genetic tests available poorly discriminate between groups with different susceptibilities, with too large misclassification for the tests to be really useful in screening. In addition, as a possible effect, which in part has been proven, there is the risk that those with the “normal” or commoner variant feel protected and do not stop smoking.^9^ This example could be expanded to the expotype. Avoidance of exposure at the population level is much more likely to lead to success than identification of high risk individuals via an expotype characterization.

## The value of choice versus structural interventions

Individualized prevention implies a key role of the individual in modifying their exposures via behavioral changes, as opposed to a role of the State in changing the distribution of risk factors in the population via taxation or prohibitions. The interference of the State with individual choices has been defined critically as an intrusion of “the nanny-State.” Such a paternalistic role has been challenged by appealing to the key principle of liberal philosophy, expressed in particular by John Stuart Mill in his *On Liberty*. For Mill and the liberal tradition, a government cannot exercise power on individuals to protect them from themselves. The only purpose for which power can be exercised on members of a community against their will, is to prevent harm to others (known as the “harm principle”). Important counter-arguments to these objections have been raised, for example that individual choices are limited by the availability of goods, their costs, and advertising and marketing policies. A healthy diet is by far much more expensive in many countries (like the UK) than a diet based on ultraprocessed food, rich in fats, salt and sugar. There are also many types of behavior linked to health that have consequences on others or on public assets. Alcohol abuse has numerous negative consequences, including road accidents and violence; the increase in diseases linked to obesity like diabetes leads to overcrowded public hospitals, etc, In brief, reducing the debate to the contrast between individual choices versus the nanny-State is a hyper-simplification, which does not consider the disproportion of power between the individual and powerful industries, a problem that did not exist at Mill’s time.

The great successes in the fight against infections in the nineteenth century derived from societal action, in particular the introduction of the sewage system, drinking water, town planning and later vaccinations. In the twentieth century enormous successes were achieved in the fight against transmissible diseases, recently in particular through the GAVI alliance.^10^ None of these preventive interventions were individualized (vaccines are administered individually, but administration does not depend on individual characteristics, except age). We have not seen similar systematic efforts targeted to non-communicable diseases, with the exceptions of taxation of cigarettes and alcohol, the prohibition of trans fatty acids in certain countries, (partial) measures to reduce air pollution and some other sporadic examples. Let us take obesity. The WHO has repeatedly called for a switch in emphasis from individual responsibility (and the view that the solution was for people to “eat less and move more”), to acknowledging that personal choices are greatly constrained by obesogenic environments which can only be addressed by governmental action. Sugar consumption is a major contributor to human obesity, but once again individual nudges to reduce intake have been unsuccessful compared to taxation. A meta-analysis indicated that taxes resulted in average reductions of 15% in sales and 18% in intake of sweet beverages.^11^ Conversely, another meta-analysis indicated that a 20% price reduction could increase fruit and vegetable purchases by 16.6%.^12^

## Conclusions

To summarize, the expotype requires proof of causality to be included into clinical practice, but the added value of the expotype may be limited, ie, correspond to a modest decrease in the NNT/NNS. An important consideration is that the medical system may not afford the extra budgets to measure the expotype at the patient’s bed. Would this be covered by the national health system budget or by an insurance? In an era of economic crisis of most health systems, the addition of an extra burden needs to be strongly justified.

More importantly, public health is largely a matter of structural interventions at the societal level, like taxation, and not only of individual responsibility. We have faced for a long time the failure of health promotion based on nudges. Naive conceptions of society, that do not consider asymmetries of knowledge and power, may underlie the emphasis of the exposome approach on the individual.

The proposal of extending precision therapy to precision prevention should not let us forget about the many opportunities for prevention at the population level. Population prevention is cheaper, it usually addresses several diseases with a single intervention (think of smoking or air pollution) and does not need to be replicated at each generation like cure.^10,13^

## Data Availability

Not relevant.

## References

[1] Roberts MC, Holt KE, Del Fiol G, Baccarelli AA, Allen CG (2024). Precision public health in the era of genomics and big data. Nat Med.

[2] National Institutes of Health (2025). The Future of Health Begins with all of us.

[3] Sarigiannis DA (2017). Assessing the impact of hazardous waste on children’s health: The exposome paradigm. Environ Res.

[4] Lloyd-Jones DM, Leip EP, Larson MG (2006). Prediction of lifetime risk for cardiovascular disease by risk factor burden at 50 years of age. Circ Am Heart Assoc.

[5] Chou R, Dana T, Blazina I, Daeges M, Jeanne TL (2016). Statins for prevention of cardiovascular disease in adults: Evidence report and systematic review for the US preventive services task force. JAMA.

[6] Richardson A (2001). Screening and the number needed to treat. J Med Screen.

[7] Vineis P, Schulte P, McMichael AJ (2001). Misconceptions about the use of genetic tests in populations. Lancet.

[8] Rose G (2001). Sick individuals and sick populations. Int J Epidemiol.

[9] Sanderson SC, O’Neill SC, Bastian LA, Bepler G, McBride CM (2010). What can interest tell us about uptake of genetic testing? Intention and behavior amongst smokers related to patients with lung cancer. Public Health Genomics.

[10] Bray F, Jemal A, Torre LA (2015). Long-term realism and cost-effectiveness: primary prevention in combatting cancer and associated inequalities worldwide. J Natl Cancer Inst.

[11] Andreyeva T, Marple K, Moore TE, Powell LM (2022). Evaluation of Economic and Health Outcomes Associated With Food Taxes and Subsidies: A Systematic Review and Meta-analysis. JAMA Netw Open.

[12] Huangfu P, Pearson F, Abu-Hijleh FM (2024). Impact of price reductions, subsidies, or financial incentives on healthy food purchases and consumption: a systematic review and meta-analysis. Lancet Planet Health.

[13] Vineis P, Wild CP (2017). The science of precision prevention of cancer. Lancet Oncol.

